# Follicular Fluid Growth Factors and Interleukin Profiling as Potential Predictors of IVF Outcomes

**DOI:** 10.3389/fphys.2022.859790

**Published:** 2022-06-13

**Authors:** Bouricha Molka, Bourdenet Gwladys, Bosquet Dorian, Moussot Lucie, Benkhalifa Mustapha, Cabry Rosalie, Gubler Brigitte, Khorsi-Cauet Hafida, Benkhalifa Moncef

**Affiliations:** ^1^ Reproductive Medicine, Reproductive Biology and Genetics, University Hospital and School of Medicine, Picardie University Jules Verne, Amiens, France; ^2^ Department of Immunology Laboratory, Amiens University Hospital, Amiens, France; ^3^ HEMATIM - EA4666, Jules Verne University of Picardie, Amiens, France; ^4^ HB Laboratory, Tunis, Tunisia; ^5^ Faculty of Sciences of Bizerte, Carthage University, Bizerte, Tunisia; ^6^ Department of Molecular Oncobiology, Amiens University Hospital, Amiens, France; ^7^ PERITOX laboratory, CURS, Picardie University Jules Verne, Amiens, France

**Keywords:** growth factors, interleukins, oocyte quality, repeated implantation failure, cumulative pregnancy rate

## Abstract

Growth hormone (GH) has gained attention as an anti-aging compound enhancing oocyte quality. In fact, GH is known to activate intrafollicular metabolic events for oocyte maturation. Insulin growth factor I (IGF1) is another ovarian growth factor that mediates the FSH and GH actions. Cytokines could also increase IVF outcomes. Indeed, IL-6 is a pleiotropic cytokine with multiple cellular effects that can vary based on the physiological environment. IL-6 may also play an important role in follicular development (Yang et al., J Assist Reprod Genet, 2020, 37 (5), 1171–1176). Clinical studies have been performed to explore the potential role of IL-6 in human oocyte maturation and subsequent embryonic development. To date, the answers are not conclusive. During peri-implantation, many cytokines balances are regulated like pro-inflammatory and anti-inflammatory interleukins. The pro-inflammatory properties of IL-17 and its impact on the tumor microenvironment or autoimmune diseases are characterized, but new dimensions of IL-17 activity that promotes embryo implantation are not well explored. In the search for answers, our study compared concentrations of growth factors IGF1, GH, and interleukins IL-6 and IL-17 in the follicular fluid (FF) from 140 women divided into two groups depending on bad (G1) or good prognosis (G2) and investigated the relationships between these FF components’ levels and the main parameters of IVF. GH, IGF1, and IL-6 were significantly higher for G2. For GH, it was negatively correlated to patient age and positively correlated to maturity rate and IGF1. Moreover, GH and IGF1 were correlated to the top embryo rate and cumulative pregnancy rate. Regarding IL-6, it was correlated to IGF1 level, endometrium thickness, and implantation rate. As for IL-17, it was only correlated to IL-6. Consequently, all these FF components were predictive of oocyte quality except IL-17. GH seemed to be the best biomarker of this quality.

## Introduction

Follicular fluid (FF) is considered a biological fluid translating hormonal changes happening in the microenvironment of an oocyte surrounded by its granulosa cells (GCs) (Wiener-Megnazi et al., 2004). In fact, it contains several biochemical components including cytokines, chemokines, growth factors, and steroid hormones which are involved in the process of folliculogenesis, and it changes by physiological conditions. During gonadotrophins treatment for controlled ovarian stimulation, as individual cells of the ovarian follicle respond to the administered gonadotrophins by secreting these components, it is normal that FF composition shows dynamic changes. It is, therefore, logical to think that FF can act as a predictor of IVF outcome parameters either directly or indirectly, such as oocyte maturity, fertilization rate, embryo quality, pregnancy rate, and implantation rate (Singh et al., 2016).

Moreover, FF composition also changes by aging. Indeed, the decline in successful conception decreases more rapidly after 38 years because of the decrease in ovarian reserve and the decrease on oocyte quality. Growth hormone (GH) has gained attention as an anti-aging compound which maintains oocyte quality. In fact, GH is known to enhance intrafollicular metabolic events required for oocyte maturation, such as the FSH-dependent E2 production by GC, the formation of FSH and LH receptors in GC, or the stimulation of androgen production by theca cells (Weall et al., 2015; Hou et al., 2019). Insulin growth factor I (IGF1) is another well-characterized ovarian growth factor that has been suggested to mediate the local action of FSH and GH (Ipsa et al., 2019).

Cytokines could also increase IVF outcomes. In fact, interleukin 6 (IL-6) is a pleiotropic cytokine because it has multiple cellular and tissular effects and leads to pro-inflammatory response. IL-6 may also play an important role in follicular development (Yang et al., 2020). Clinical studies have been performed to explore the potential role of IL-6 in human oocyte maturation and subsequent embryonic development, but no robust conclusion could be drawn based on the results. Some studies have shown that a higher level of IL-6 correlated with worse quality of embryos, and patients were less likely to get pregnant (Semeniuk et al., 2018). In contrast, other studies have shown that high levels of IL-6 in the FF were good for oocyte maturation and were associated with increased rates of clinical pregnancy and embryo implantation (Wu et al., 2017). Indeed, during the peri-implantation period, immunological balances must be regulated including regulation of pro-inflammatory/anti-inflammatory chemokines and IL17/regulatory T cells (Ali et al., 2021). The pro-inflammatory properties of IL-17 and its impact on tumor microenvironment or autoimmune diseases are extended, and characterized but new dimensions of IL-17 activity that promote embryo implantation are not well explored (Zhao et al., 2021).

Most of these studies on follicular growth factors and interleukins, however, were interested in good prognosis patients to demonstrate FF components and IVF outcomes correlations and divided their population based on clinical pregnancies. However, these studies are made with the uncertainty of whether there are FF abnormalities for patients with fertilization failure or repeated implantation failure (RIF).

These previous findings raised some questions. Is the intrafollicular concentration of a given cytokine or growth factor associated with oocyte quality? Are the abnormal FF patterns reflected by an easily detectable oocyte or embryo characteristic (such as fertilization failure of embryo morphology)?

In the search for answers to these questions, the present study compared mean concentrations of growth factors (IGF1 and GH) and interleukins (IL-6 and IL-17) in FF in 140 women divided into two groups depending on good or bad prognosis. We have investigated the relationships between these FF component levels and the main parameters of controlled ovarian stimulation, embryo laboratory outcomes, and pregnancy outcomes.

## Materials and Methods

### Study Setting

We conducted a prospective and longitudinal study in the reproductive medicine center at Amiens Picardie University Hospital (Amiens, France) from June 2021 to November 2021 in collaboration with the research laboratory and immunology laboratory. All the couples participating in an *in vitro* fertilization (IVF) or intra cytoplasmic sperm injection (ICSI) program in this period, were included. Criteria of inclusion were as follows: patients aged between 19 and 42 years, husband aged below 46 years, first or second attempt, and number of oocytes retrieved more than or equal to 2. Criteria of exclusion were severe alteration of sperm in terms of the number below 10 million/ml or progressive motility below 20% at the day of fertilization, oocyte receivers as the follicular fluid of donors doesn’t reflect endometrial microenvironment of receivers, endometriosis patients, and patients with polycystic ovary syndrome. Based on the ethical committee rules of our institution, this study wasn’t approved by an ethics committee as only FF, considered as biological waste after oocyte pick-up, was used for analysis.

### Ovarian Stimulation and IVF Protocols

Two controlled ovarian stimulation (COS) protocols were used: a gonadotropin-releasing hormone (GnRH) long agonist protocol and a GnRH antagonist protocol.

The long agonist protocol with a GnRH agonist (triptorelin acetate: Décapeptyl^®^, Ipsen Pharma, France; 0.1 mg per day for 14 days, starting in the midluteal phase), followed by the administration of recombinant human follicle-stimulating hormone (rFSH: Puregon^®^, Organon, France, or Gonal-F^®^, Merck Serono SAS, France) or human menopausal gonadotropin (HMG, Menopur^®^, Ferring, France), in combination with Décapeptyl^®^ (0.05 mg per day).

In the antagonist protocol, rFSH was administered subcutaneously each day from day 2 of the cycle until a 14 mm dominant follicle was detected. Cetrorelix acetate (Cetrotide^®^, Merck Serono, France; 0.25 mg per day) was then administered daily until the recombinant human chorionic gonadotropin (rhCG) day (Ovitrelle^®^, Merck Serono SAS).

Patients were monitored clinically using transvaginal pelvic ultrasound and assays for estradiol, progesterone, and luteinizing hormone. The rFSH/HMG dose level was adjusted according to the follicular growth measured during the monitoring phase.

When at least three follicles had reached a diameter of more than 16 mm, a 250 μg dose of rhCG was administered. Ovulation was triggered with triptorelin (0.2 mg Decapeptyl) if a freeze-all cycle has been programmed. Oocytes were retrieved 36 h after hCG administration, via ultrasound-guided transvaginal follicular aspiration. Cumulus cells were enzymatically denuded from the oocyte complexes 38 h after the rhCG administration. All oocytes were used for IVF or ICSI according to standard protocols. Fertilization was assessed 16–18 h after sperm injection. The morphology was assessed according to the Istanbul consensus criteria (for day 2/3 embryos) (Alpha Scientists in Reproductive Medicine and ESHRE Special Interest Group of Embryology, 2011) or Gardner’s criteria (for blastocysts) (Gardner and Schoolcraft, 1999). Intrauterine transfer of embryos has been performed if a good embryo quality (morphological aspect and kinetics of development) was observed. The day of transfer and the number of embryos transferred were defined according to the history of the couple and embryo quality. It was a single or a double embryo transfer for the early cleavage stage (day 3) or a single blastocyst transfer (day 5).

Progestin (Utrogestan^®^ 400 mg, Besins International, France) was used for luteal phase support. Pregnancy was defined as a serum hCG level≥100 IU/L 14 days after embryo transfer.

### Study Groups

Our population was a representative sample. We divided it into two groups:

Group 1 (bad prognosis): women aged≥ 35 years or attempt with embryo development failure or RIF antecedent (n = 72). Embryo development failure was defined by fertilization failure: absence of two pronuclei in all oocytes after 16 h of microinjection, or by poor embryo quality. RIF was defined by the absence of clinical pregnancy after three cumulative top embryos transfer at day 3 or two cumulative blastocysts transfer at day 5.

Group 2 (good prognosis): women aged <35 years, first or second attempt (n = 68).

### Preparation of FF Samples

FF samples were collected from 140 women. Following oocyte pick-up, each patient’s remaining FF samples were pooled from a syringe that contains the most oocytes. Though few studies have also focused on monodominant follicles (fluid obtained from a single lead follicle), such estimations may not truly reflect granulosa or thecal cell production (Mehta et al., 2013a). Therefore, in each cycle, we estimated GH, IGF1, IL6, and IL17 levels in FF. Recommended nonextraction method was used for the estimation of IGF1 and GH, as the extraction method has been reported to involve interference due to binding proteins (Roussi et al., 1989). After cell removal by centrifugation (2000 g, 10 min) the supernatant was recovered, stored at -80 °C, and thawed immediately prior to analysis.

The concentrations of pro-inflammatory interleukins (IL-6, IL-17) and growth factors (IGF1, GH) were determined by ELISA.

IGF1 (IGF1 BIOTECHNE KIT) and GH (GH BIOTECHNE KIT) levels were assessed according to manufacturer recommendations and using a DS2 system for optic density determination (DYNEX, MAGELLAN BIOSCIENCES, United States).

IL-6 (Simple Plex Human IL-6 Cartridge) and IL-17 (Simple Plex Human IL-17/IL-17A Cartridge), levels were assessed according to manufacturer recommendations and using a full automated Ella System (R&D SYSTEMS a Bio-Techne Brands, United States).

### Statistical Analysis

All statistical analyses were performed with SPSS 25. Continuous data were presented as mean and standard deviation (SD) by descriptive statistics, and categorical data were given as percentages (%). Intergroup differences were probed with a Mann–Whitney test (for quantitative variables) or a Chi-square test, Fisher’s exact test, or Student’s t-test (for qualitative variables). The threshold for statistical significance was *p* < 0.05. Statistics results were adjusted by age using one-way ANCOVA adjustment at a level of 0.05.

The expression of these molecules was subjected to multivariate analysis for the identification of major predictive markers of oocyte and embryo quality. The correlative study between FF components and IVF outcomes were probed with the Pearson coefficient, if the normality assumption is not met for some variables, we used Spearman coefficient. The receiver operating characteristic (ROC) curve was applied to determine the best cutoff point for the discrimination between these components and oocyte quality in these women. The intra- and inter-assay coefficients of variation were below 10% in all cases.

## Results

Patients in Group 1 (bad prognosis) were significantly older. Both groups were comparable in terms of anti-Müllerian hormone (AMH), IVF or ICSI repartition, agonist or antagonist protocol repartition, and E2 level at trigger day, and day’s number of stimulation (Table 1). Oocyte number was significantly higher for Group2 (10.78 ± 5.44 vs. 8.91 ± 5.04, *p* = 0.04). A comparative analysis of the two groups in terms of oocytes parameters showed a significantly higher maturation rate for Group2 (81.9 vs. 69.54, *p* = 0.004), however, immaturity and degeneration rates were comparable (Table 2).

**TABLE 1:** Descriptive analysis of the two groups.

Parameters	Patient age	AMH	FIV/ICSI	Ago/Antag	E2 trigger day	Day stim
GROUP1(n = 72)						
Mean ± SD	36.01 ± 4.22	2.74 ± 2.24	6.9/93.1%	13.8/86.1%	2221.2 ± 835	10.18 ± 2.09
Min-max	36–42	0.2–13.1	—	—	1080–41877	7–13
GROUP2(n = 68)						
Mean ± SD	29.1 ± 3.59**	3.62 ± 3.52	14.7/85.3%	20.6/79.4%	2199.3 ± 1045	9.77 ± 1.35
Min-max	19–35	0.7–9	—	7–13	812–3895	7–13

** For values within column *p* < 0.01; AMH (ng/ml): anti-Müllerian hormone; Ago: agonist protocol; Antag: antagonist protocol; day stim: number of days for stimulation.

**TABLE 2:** Comparative analysis of the two groups in terms of oocyte parameters.

Parameters	Maturation %	Immaturity %	Degeneration %
GROUP1(72)			
Rate%	69.45 (n = 345)	31.52 (n = 152)	6.48 (n = 30)
GROUP2(68)			
Rate%	81.9** (n = 615)	34.56 (n = 263)	2.76 (n = 23)

*p* = 0.004.

All 140 patients underwent oocyte retrieval, and oocytes were obtained. 101 patients received a fresh embryo transfer. For Group1, 8 patients had blastocyst transfer and the rest had a single (56 patients) or double embryo transfer (8patients) on day 3. For Group2, 15 patients had blastocyst transfer and the rest had a single (44patients) or double embryo transfer (9 patients) on day 3. For both groups, 39 patients canceled transfer due to a contraindication of embryo transfer in the context of uncontrolled ovarian hyperstimulation syndrome (OHSS), thin endometrium, or poor embryo quality. 11 patients from 39, who had a freeze-all strategy, have already had a frozen embryo transfer with a substituted cycle for endometrium preparation. And 14 patients, who have already had a fresh transfer, had a second transfer by the use of their surplus frozen embryos with also a substituted cycle.

There were no differences between the two groups in terms of fertilization, top embryos, and freezing rates. Ongoing pregnancy rate (OPR) by transfer was higher in Group2 (20.1% vs. 30.5%; *p* = 0.05). The blastulation rate was significantly higher in Group2.

Freeze-all cycle rates were higher for Group2 (29.41% vs. 16.66%, *p* = 0.04). Regarding cumulative pregnancy rate (CPR), it was defined as fresh and frozen embryo transfer in two consecutive cycles. It was significantly higher for Group2 (52.1% vs. 28.2%, *p* = 0.027) (Table 3).

**TABLE 3:** Comparative analysis between the two groups in terms of IVF outcomes.

Parameters	Fertilization%	Top E	Blast%	freezing%	Implant%	OPR%	CPR%
GROUP1 (72)						
Rate%	77.1 (n = 276)	21.48 (n = 55)	14.1(35)	21.6(52)	17.1	20.5 (15)	28.2 (20)
GROUP2(68)						
Rate%	80.4 (n = 498)	27.56 (101)	33.6(123)	28.6(105)	26.2	30.1 (20)	52.1 (34)
P	—	—	0.002	—	—	—	0.027

*p* ≥ 0.05; Top E = top embryos rate; Blast = blastulation; Implant = implantation; OPR , ongoing pregnancy rate; CPR , cumulative pregnancy rate.

When mean follicular concentrations of substances measured in FF samples obtained during a single treatment attempt were compared between the two groups, the second group showed significantly higher values for GH (1.14 vs. 2.3, *p* = 0.001), IGF1 (17.71 vs. 44.78, *p* = 0.001) and IL-6 (10.35 vs. 69.58, *p* < 0.05) (Table 4).

**TABLE 4:** Concentrations of growth factors and interleukins in FF.

Parameters	IGF1 1 (ng/ml)	GH (ng/ml)	IL-6 (pg/ml)	IL-17 (pg/ml)
GROUP1 (n = 72)				
Mean ± SD	17.71 ± 20.15	1.14 ± 1.08	10.35 ± 7.9	0.25 ± 0.43
Min-max	0.63–100.8	0.2–6.15	2.12–87.3	0–1.89
GROUP2 (n = 68)				
Mean ± SD	44.78 ± 57.58	2.3 ± 2.31	69.58 ± 436.8	0.23 ± 0.44
Min-max	5.2–281 (*p* = 0.001)	0.49–12.3 (*p* = 0.001)	0–3391 (*p* = 0.05)	0–1.67


IGF1 = insulin-like growth factor 1; GH , growth factor; IL-6 , interleukin 6; IL-17 , interleukin 17.

**FIGURE 1:**
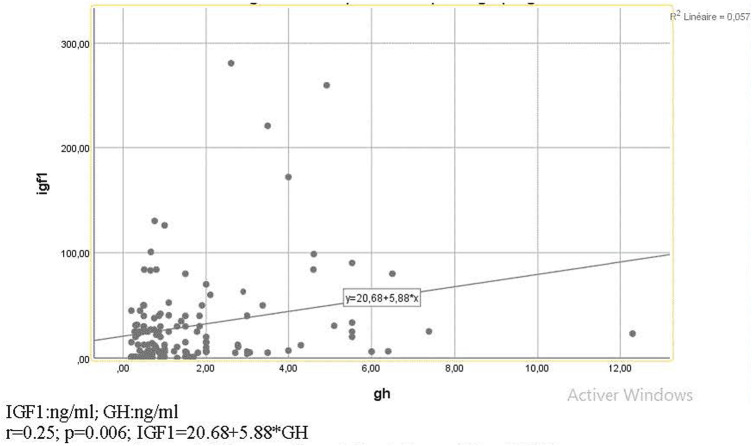
Diagram of correlation between GH and IGF1.

**FIGURE 2:**
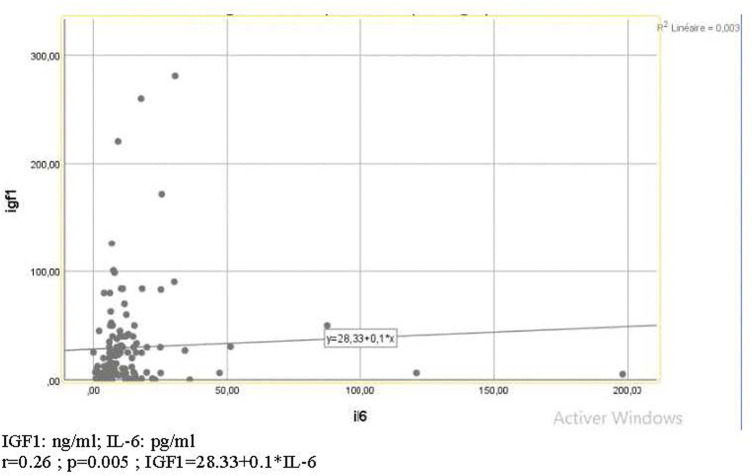
Diagram of correlation between IL-6 and IGF1.

**FIGURE 3:**
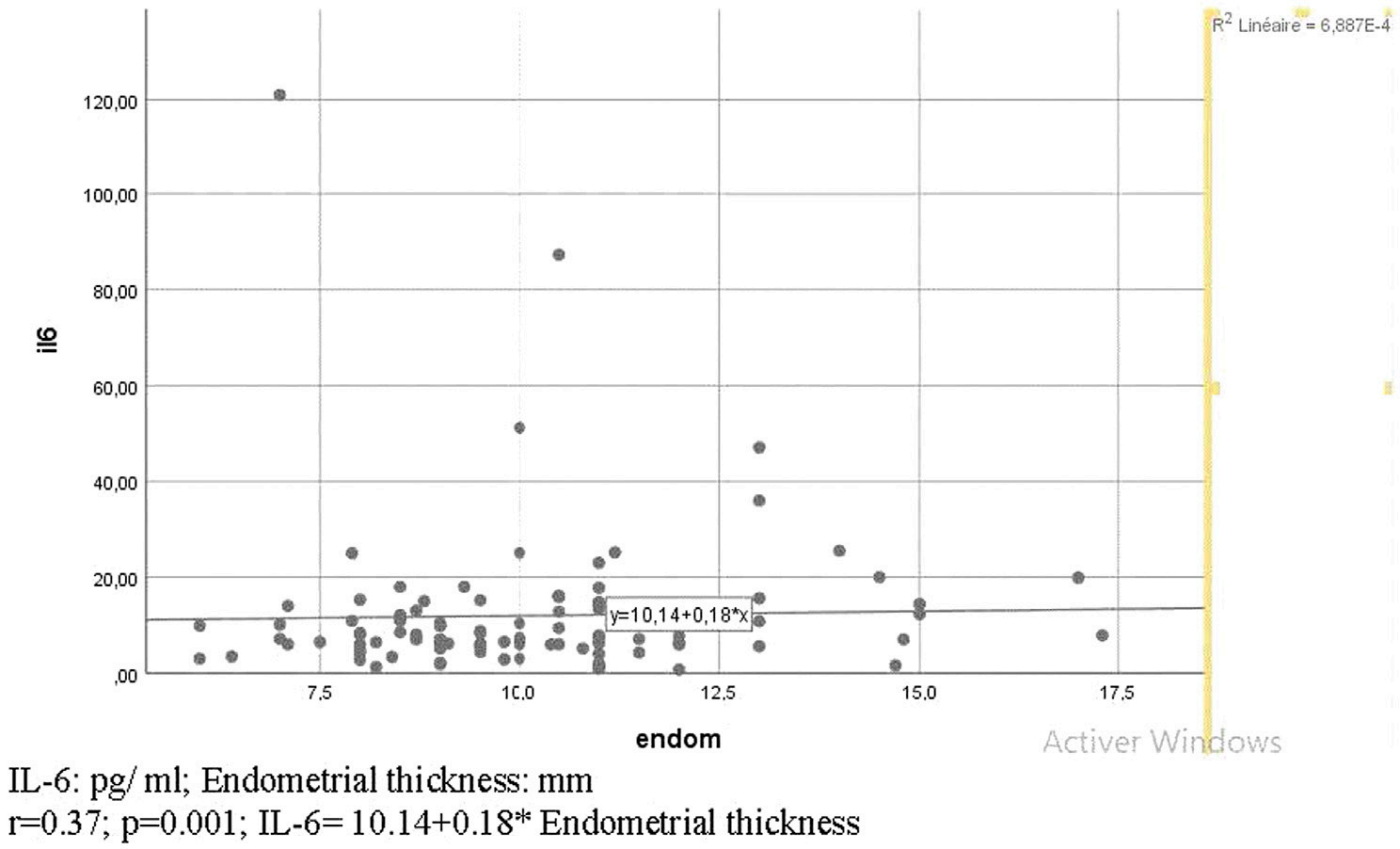
Diagram of correlation between IL-6 and endometrial thickness.

Many correlations were significant (Figures 1,2, Table 4, Figure 3), consequently, we have determined thresholds for GH, IGF1, and IL-6. GH level was significantly correlated with the implantation rate of ongoing pregnancy and the cutoff of GH was 1.01 ng/ml (AUC = 0.66 and *p* = 0.029). Regarding IGF1, it was associated with CPR and IGF1 threshold>51.26 ng/ml (AUC 0.73; *p* = 0.001). As for IL-6, the ROC curve between this interleukin and implantation rate of ongoing pregnancy found an IL-6 cutoff >9.85 pg/ml (AUC 0.6; *p* = 0.04).

## Discussion

Our comparative study between the two groups has shown significantly higher rates in terms of oocyte maturation, blastulation, and cumulative pregnancy (CPR). This suggests a better oocyte quality for good prognosis patients which is reflected also by a significantly higher GH, IGF1, and IL-6 levels.

Our correlative study between FF components and ICSI outcomes has found many significant correlations.

### GH and IVF Outcomes

#### Oocyte and Embryo Quality

GH was significantly higher for Group 2 and negatively correlated with age. It was proven that GH receptor expression within GCs of human Graafian follicles declines with age which leads to defects in chromatid separation, chromosome decondensation (Regan et al., 2012), and spindle detachment causing chromosomal misalignment (Liu and Keefe, 2002). Therefore, the oocyte aneuploidy rate increases with age, which leads to oocyte quality deterioration and infertility as the cause of decreased fertility are mainly genetic (Severance and Latham, 2018).

We have found a significant correlation between GH and embryo morphology. In fact, GH is known to enhance intrafollicular metabolic events required for oocyte maturation by enhancing the expression of genes associated with meiotic progression and embryo development, such as aurora kinase A 13, protein disulfide isomerase family A member, leucine-rich repeat and Ig domain containing 2, and centromere protein J (Li et al., 2019). Moreover, GH not only accelerates meiotic progression by stimulating these genes expression, but also by providing energy for meiotic spindle formation. In fact, it improves mitochondrial function when added during ovarian stimulation and increases ATP level, so it increases the number of mature oocytes and embryos obtained (Bassiouny et al., 2016). Furthermore, it regulates the redox homeostasis balance of the cellular environment, consequently, it enhances cytoplasmic competence and embryo quality (Gong et al., 2020).

#### Implantation Rate and CPR

GH was significantly correlated to implantation rate and CPR (Table 5). Indeed, Panel et al. have found that the lack of GH receptor and GH-binding protein is associated with a decreased level of GH in FF which leads to a significant reduction in follicular development and oocyte number after gonadotrophin stimulation (Ho et al., 2017). Furthermore, several meta-analyses showed that the addition of GH to gonadotrophins significantly increased the pregnancy rate and live birth rate for poor responders by enhancing oocyte number (Kolibianakis et al., 2009). Ye Chen et al. have proved GH efficacy in 22 patients with RIF (G1) by comparing them to another RIF group (n = 20) without GH treatment (G2). They found in G1 higher levels of micro RNA GH receptor which means higher levels of GH in FF. They conclude that GH must be added for RIF patients because it increases significantly IVF clinical outcomes (Chen et al., 2018).

**TABLE 5:** Multivariate linear regressions matrix comparing FF biomarkers and IVF outcomes.

Parameters	IGF1 (ng/ml)	GH (ng/ml)	IL-6 (pg/ml)	IL-17 (pg/ml)
Patient age	—	Age-GH *p* = 0.009	—	—
OR and 95%CI	—	1.52; 1.13–2.05	—	—
Endometrial thick	—	—	E-IL-6 *p* = 0.001	—
OR and 95%CI	—	—	1.5; 1–2.1	—
Top embryo%	Top embryo-IGF-1	Top embryo-GH	—	—
	*p* = 0.03	*p* = 0.02	—	—
OR and 95%CI	1.35; 1.14–1.61	1.29; 0.99–1.68	—	—
Implantation %	—	impl-GH *p* = 0.02	impl-IL-6 *p* = 0.04	—
OR and 95%CI	—	1.4; 1–1.9	1.1; 0.99–1.2	—
CPR	CPR-IGF-1 *p* = 0.03	CPR-GH *p* = 0.001	—	—
OR and 95%CI	1.1; 1–1.3	3.63; 1.6–8.2	—	—

IGF1 = insulin-like growth factor; GH , growth factor; IL-6 , interleukin 6; IL-17 = interleukin 17; Impl = implantation; E = endometrial thickness_: correlation was not significative.

Moreover, Cui et al. reported that GH improves endometrium thickness for cycles with frozen embryo transfer at day 3, especially for patients with thin endometria antecedent that contraindicated fresh transfer. In fact, it up-regulates genes of endometria receptivity. Consequently, it improves implantation and clinical pregnancy rates by promoting endometria proliferation and vascularization (CuiA et al., 2018).

#### GH and IGF1

GH and IGF1 were significantly correlated (Figure 1, Table 5). That can be explained by the fact that GH can induce IGF1 secretion in the liver and the ovary (Dosouto et al., 2019). They both have actions in common like stimulating ovarian folliculogenesis (Ipsa et al., 2019). GH also mediates FSH action on GC through enhancing local IGF1 synthesis. In turn, IGF1 increases in an autocrine way gonadotropin action at GC and theca cells by mediating aromatase activity and follicular estrogen production (Ipsa et al., 2019). Thus, GH increases IGF1 intra-ovarian production, which is considered important for oocyte maturation (Stoecklein et al., 2021).

### IGF-I and IVF Outcomes

#### Oocyte and Embryo Quality

Our study not only reports significantly higher levels of FF IGF1 in good prognosis versus bad prognosis cycles but also found a strong, direct correlation of FF IGF1 with the top embryo rate. A very recent study has found that IGF1 activates E-cadherins molecules that are responsible for cell division and trophectoderm cells survival (Stoecklein et al., 2021). In fact, IGF1 works through E-cadherins activation in order to stimulate mitogen-activated protein kinases signaling cascade and phosphatidyl-inositol three kinase pathways, both of which are involved in cell clustering and cell-renewal (Bedzhov et al., 2012).

#### Cumulative Pregnancy Rate (CPR)

IGF1 was significantly correlated with CPR. Threshold value of FF IGF1 for CPR was >51.26 ng/ml (receiver operating characteristics AUC = 0.73, *p* = 0.001). Consistent with our study, Bindu et al. have found a higher cutoff >58.50 ng/mg protein (AUC = 0.85; *p* = 0.001) probably because they include only good prognosis patients (Mehta et al., 2013b).

Another study indicated that the embryo influences the intra uterine environment during the invasion phase by the mediation of IGF1. In fact, transferred blastocysts in a culture medium enriched by IGF1 increases endometrium thickness significantly (Yuan et al., 2017). Furthermore, the activation of phosphatidyl-inositol three kinase by IGF1 will initiate trophoblast invasion and the activation of E-cadherins by this biomarker in conjunction with metalloproteinases ADAM 10 will activate pro-inflammatory cytokines obligatory for peri-implantation (Peron et al., 2018). IGF1 also participates in the modulation of the secretion of matrix metalloproteinases (MMP) and their specific inhibitors; the tissue inhibitors of matrix metalloproteinases (TIMP), which are also involved in the human trophoblast cells invasion, as well as follicular development in oocyte level (Benkhalifa et al., 2018). On the other side, IGF2 is more likely associated with implantation rate than IGF1. Studies have shown that IGF2 is expressed by the trophoblast during the invasion phase, and the fixation of IGF2 on its endometrium receptors will influence the development of vessels near implantation sites. This suggests that IGF2 may contribute to the formation of decidual stromal cells (Yuan et al., 2017). The association between IGF1 and CPR probably suggests that intra-individual variability of IGF1 between cycles could be nonexistent if close cycles are performed. Indeed, patients who had high levels of IGF1 measured during the stimulated cycle for oocyte pick-up but scheduled for FET were then pregnant.

### IL-6 and IVF Outcomes

#### IL-6 and IGF-I

IL-6 was significantly correlated to IGF1 (Figure 2, Table 5). Indeed, it plays similar roles in regulating the proliferation and apoptosis of GC. Furthermore, the addition of these cytokines to the culture medium has increased the blastulation rate by stimulating cell division and survival rate after cryopreservation by enhancing the resistance to thermal shock (Stoecklein et al., 2021). Their action seems to be synergistic because their combination in a culture medium improves the embryo number by increasing the cell number and embryo quality (Boldeanu et al., 2020).

#### Oocyte and Embryo Quality

As IL-6 and IGF1 were correlated, IL-6 could be associated with oocyte and embryo quality. In literature, high levels of FF IL-6 increased the percentage of preantral follicular survival rate (Yang et al., 2020). A previous study has shown that the disruption of GP130, an expressed signal-transducing receptor shared by IL6 and leukemia inhibitory factor, can cause serious oocyte and embryo degeneration (Kocyigit and Cevik, 2015). Other studies have confirmed these findings by showing that the addition of IL-6 to the culture medium significantly decreased the apoptotic cell rate (Molyneaux et al., 2003).

#### 3 Endometrium Thickness and Implantation Rate of OPR

Our study finds a significant correlation between IL-6 and endometrium thickness (Figure 3, Table 5). Moreover, there was also a significant correlation between IL-6 and implantation rate. In fact, Shafat et al. have demonstrated in a recent review that during early implantation, anti-inflammatory interleukins like TGF-B and IL-10 decreased, and the inflammatory responses synchronize the endometrial decidualization (Ali et al., 2021). Indeed, inflammatory cytokines like TNFα and IL1β, IL6 are released from the embryo and stromal cells. They constitute the signals between the mother and the embryo during early implantation and they initiate trophoblast invasion (Shen et al., 2009). IL-6 is also involved in the regulation of TIMP-1 secretion by theca, GC, ovary surface epithelium, corpora lutea, blood vessels, and the oocyte which could produce the TIMP (Zhu et al., 2012; Bianchi et al., 2016). For this, and as for example, a down-regulation of TIMP-1 could play a critical role in the ovulation process and an altered MMP/TIMP balance could lead to an early regression of the corpus luteum with consequent exhaustion of progesterone and estradiol synthesis, thereby impacting embryo implantation (Goldman and Shalev, 2004). In addition, IL-6 stimulates TIMP-1 (Mori et al., 2016) that is known to increase in FF of the blastocyst to facilitate implantation and to be correlated to metalloproteases 10 which is necessary for implantation by the inflammatory potential (Sanchez et al., 2004; Zhang et al., 2004; Plaks et al., 2013). The present study confirmed that high levels of FF IL-6 can significantly improve implantation rate, which is also consistent with the work of Dominguez F et al. In their study, blastocysts that secreted more IL-6 into the culture medium had a significantly higher implantation rate in comparison with blastocysts that did not (Dominguez et al., 2015).

### IL-17 and IVF Outcomes

#### 1 Oocyte and Embryo Quality

We found similar IL-17 levels between the two groups (FF level = 0.23 pg/ml). In fact, as we excluded patients with endometritis, endometriosis, or polycystic ovary syndrome (PCOS), the expression of IL-17 was comparable although it was a different prognosis (Berridge and Irvine, 1989; Wang et al., 2019). A recent study, analyzing FF of infertile patients found an IL-17 level higher in the FF of endometriosis and PCOS women than in the control group (FF level = 1.25 pg/ml). Indeed, they have a higher rate of oxidative stress that plays a central role in syndrome pathogenesis and oocyte mitochondrial dysfunction (Reiter et al., 2002). This oxidative stress will in turn increase the IL-17 level (Xu et al., 2018). Consequently, a too high IL-17 level could play an important role in damaging egg quality. In our study, as the IL-17 level was three times lower than levels in this last study, it could be correlated with embryo quality by IL-6 mediation.

#### 2 IL-6 and IL-17

IL-17 belongs to pro-inflammatory cytokines secreted by CD4^+^ T helper 17 (Th17) cells. It acts on the IL-17 receptor to initiate an inflammatory response through pro-inflammatory cytokines from decidual cells (Unfer et al., 2011). Indeed, we found a significant correlation between IL17 and IL6. Recent studies show that IL- 17-IL-6 axis is a new topic and that IL-17 activates inflammation but also repairs tissue through IL-6 (Valmori et al., 2010). In fact, in the cancerology field, it was observed that IL-17 induces IL-6 production through a combination of transcriptional and posttranscriptional changes. They both, induce micro RNA metabolism that encodes inflammatory factors like TNF (Zhao et al., 2020). It was also reported that in psoriasis patients, IL-17 will then synergize with TNF to intensify inflammatory response (Chiricozzi et al., 2011).

#### 3 Implantation Rate of OPR

Our study has found an indirect correlation between IL-17 and implantation rate. In fact, IL-17 as a mediator of IL-6 and TNF may confer a suitable microenvironment for trophoblast invasion. Another study has shown that IL-17 does not only increase implantation by mediating IL-6 activity, but it also mediates prostaglandin E2 action in the materno-fetal cross talk (Michimata et al., 2002). Furthermore, it was shown that in lung adenocarcinoma, IL-17 induces angiogenic factors through stimulating vascular endothelial growth factor (VEGF) which is primordial for vascular development in implantation sites (Huang et al., 2016). In addition to the impact on the endometrial microenvironment during implantation, recent studies have discovered new dimensions of IL-17 activity like activating metalloproteinase9 production related to TIMP1 expression (Jovanovic et al., 2001). IL-17 exhibits also lower levels during implantation which is consistent with our study (around 0.23 pg/ml for the two groups) and begins to increase from the second trimester to reach 0.8 pg/ml and to maintain a healthy pregnancy (Martínez-García et al., 2011).

### Perspectives

The measure of IGF2 in FF could also be a predictor of embryo implantation. While IGF1 is the mediator of cell proliferation, IGF2 regulates implantation window and perinatal development (Yuan et al., 2017). Moreover, it could be interesting to study the intra-individual variability of IGF1 and GH between successive ovarian stimulation cycles as they were correlated to CPR in our study or to compare biomarkers follicular levels between agonist and antagonist protocols. Furthermore, studying TGF-B and IL-10 as anti-inflammatory interleukins during the implantation period could be very interesting as they exhibit lower levels to maintain the balance of inflammatory/anti-inflammatory cytokines. Adding growth factors or IL-6 to the culture medium may constitute a therapeutic solution for patients with RIF or with a failure of embryo development as these cytokines not only act on GC but also on cell division. A low level of IL-6 may be a new indication for freeze-all cycles as it is a predictor of implantation failure. Finally, making a new kit for measuring these follicular biomarkers that are correlated with oocyte and embryo quality, could be a new noninvasive method that predicts the implantation potential of each embryo.

## Conclusion

We conjectured that the FF microenvironment with its growth factors and interleukins may predict embryo development and may dictate the implantation potential of the ensuing embryo. Therefore, evaluating levels of biochemical biomarkers in FF may be a noninvasive approach than extrapolating data from invasive methods like embryo biopsy. Moreover, we get an assessment of embryo quality by studying GC replication as we collect FF from which there were oocytes. Our hypothesis was justified by a direct correlation obtained in our study between FF growth hormone levels (GH and IGF1) and embryo quality especially CPR and between IL-6 and implantation rate of OPR. Our study has also determined an IL-17 average during the implantation period that is original and must be confronted with other studies.

## Data Availability

The original contributions presented in the study are included in the article/Supplementary Material, further inquiries can be directed to the corresponding author.
